# Which Comes First, Nonalcoholic Fatty Liver Disease or Arterial Hypertension?

**DOI:** 10.3390/biomedicines11092465

**Published:** 2023-09-05

**Authors:** Julia A. Golubeva, Anna F. Sheptulina, Anastasia Yu. Elkina, Ekaterina O. Liusina, Anton R. Kiselev, Oxana M. Drapkina

**Affiliations:** 1 Department of Fundamental and Applied Aspects of Obesity, National Medical Research Center for Therapy and Preventive Medicine, 101990 Moscow, Russia; 2 Department of Therapy and Preventive Medicine, A.I. Evdokimov Moscow State University of Medicine and Dentistry, 127473 Moscow, Russia; 3 Department of Intermediate Level Therapy, Saratov State Medical University, 410012 Saratov, Russia; 4 Coordinating Center for Fundamental Research, National Medical Research Center for Therapy and Preventive Medicine, 101990 Moscow, Russia

**Keywords:** non-alcoholic fatty liver disease, hypertension, steatohepatitis

## Abstract

Non-alcoholic fatty liver disease (NAFLD) and arterial hypertension (AH) are widespread noncommunicable diseases in the global population. Since hypertension and NAFLD are diseases associated with metabolic syndrome, they are often comorbid. In fact, many contemporary published studies confirm the association of these diseases with each other, regardless of whether other metabolic factors, such as obesity, dyslipidemia, and type 2 diabetes mellites, are present. This narrative review considers the features of the association between NAFLD and AH, as well as possible pathophysiological mechanisms.

## 1. Introduction

Non-alcoholic fatty liver disease (NAFLD) and arterial hypertension (AH) are common noncommunicable diseases in the global population. To date, the impact of NAFLD extends far beyond the liver; its association with an increased risk of cardiovascular disease (CVD), both in isolation and as part of the metabolic syndrome, has been proven [1]. The relationship between CVD and NAFLD is also explained by the commonality of risk factors underlying the development of these diseases. In addition to already known risk factors (abdominal obesity [2], high blood cholesterol [3], metabolic disorders [4], etc.), data have been obtained on other factors that can trigger the development and progression of both NAFLD and CVD [5]. Among these, we should mention an increase in serum levels of uric acid [6], C-reactive protein, interleukin (IL)-6 [7], fibrinogen, von Willebrand factor, and plasminogen activator inhibitor-1 [8], as well as an augmented thickness of epicardial fat [9] and intima-media complex [10]. AH is included in the criteria for metabolic syndrome [11]. It accompanies NAFLD quite frequently and is a major risk factor for CVD [12].

Although NAFLD is not included in the criteria for metabolic syndrome [13,14], a growing body of evidence indicates an association between AH and NAFLD and supports the concept that NAFLD may be considered as the hepatic manifestation of metabolic syndrome [5]. In our narrative review, we consider the research results, features, and possible pathophysiological mechanisms explaining this association.

## 2. Methods

The objective of this literature review was to identify evidence that demonstrates the relationship between the development and progression of NAFLD and arterial hypertension with a focus on the most recent data (published within the past 5 years, 2018–2023). Papers published before 2018 were included only when they provided information critical for the topic of the present literature review. In this regard, we searched the PubMed electronic database on 25 May 2023 using the following PubMed filters:

- Article type: Classical Article, Clinical Study, Clinical Trial, Comparative Study, Con- trolled Clinical Trial, Multicenter Study, Meta-Analysis, Observational Study, and Randomized Controlled Trial, Preprint;
- Species: Humans, Other Animals;
- Article language: English;
- Age: Adult;
- Publication date: 5 years.

Articles outside of the PubMed search were also included, if relevant.

Articles were included in the full-text narrative review in cases where they reported on disease burden, epidemiology, and pathogenic mechanisms of the relationship between NAFLD and arterial hypertension.

## 3. Prognoses of Patients with NAFLD and AH

Regardless of common risk factors, NAFLD is associated with a higher probability of developing CVD and their complications. The latter include atherosclerotic manifestations (thickening of the intima-media, endothelial dysfunction, and increased arterial stiffness), left ventricular hypertrophy, calcification of the aortic valve and coronary arteries [15,16]. The comorbidity of NAFLD and AH significantly increases the risk of atherosclerosis compared to what is observed in the presence of only one of these diseases [17].

Epidemiological studies suggested that the simultaneous presence of NAFLD and AH, regardless of other factors, significantly aggravates the prognosis of patients and increases the likelihood of cardiovascular complications [18]. A large study involving a Chinese cohort established that patients with comorbid AH and NAFLD had a higher risk of CVD mortality vs. those with NAFLD alone. In addition, the risk of death was higher in patients with NAFLD and uncontrolled AH (hazard ratio [HR] 2.36, 95% confidence interval [CI] 1.36–4.10, *p* < 0.01). After adjusting for confounding factors (presence of diabetes mellitus, body mass index [BMI], age, gender, and smoking status), statistically significantly higher mortality rate was still observed [19]. Also, during a 13-year follow-up period for over 70,000 patients with NAFLD, it was noted that the presence of high normal blood pressure (BP) (in the range of 120/80–139/89 mmHg) increased the risk of developing CVD and CVD-related mortality 1.5-fold when compared with the group with normal BP [20]. Hence, even early-onset hypertension in individuals with NAFLD aggravates the prognosis of patients.

A prospective study of 271,906 patients with NAFLD established that the likelihood of NAFLD progressing to cirrhosis or developing hepatocellular carcinoma increased with each additional component of the metabolic syndrome: e.g., in the presence of AH, the risk increased by 59% [21]. This may be explained by the fact that frequent co-occurrence of metabolic conditions and their interplay may contribute to liver disease progression and hepatocarcinogenesis through the influence on common pathophysiological mechanisms, such as oxidative stress, lipotoxicity, cytokine disbalance, etc. [22]. However, interpreting negative impact of the comorbidity of these diseases seems problematic due to the high prevalence of concomitant confounding factors, such as obesity, diabetes mellitus, and hyperlipidemia. There is evidence that the presence of visceral fat rather than an excess of total body weight per se predicts the risk of progression of steatosis to steatohepatitis and liver fibrosis [23]. For instance, it is known that, among Asian people, NAFLD is most common in patients with normal BMI and simultaneous visceral obesity (high waist circumference) [24].

It should be noted that the association between the presence of NAFLD and AH in thin patients was observed as well [24]. The results of a Chinese patient cohort study were published in 2023. They demonstrated that, even though patients had normal body weight (BMI < 25 kg/m^2^), the AH was the main cause of NAFLD development with an overall HR of 2.05 (95% CI 1.87–2.25), and five different statistical methods were used for the HR calculation to assess the relationship [25]. Therefore, it can be concluded that the relationship between NAFLD and AH exists regardless of obesity (BMI ≥ 30 kg/m^2^ for the Caucasian population [26] and BMI ≥ 25 kg/m^2^ for the Asian population [27]) and overweight (BMI ≥ 25 kg/m^2^ for the Caucasian population [26] and BMI ≥ 23 kg/m^2^ for the Asian population [27]), and the combination of these two may have a more pronounced negative impact on the patient prognosis vs. the individual effect of each of them. 

### 3.1. NAFLD as Independent Risk Factor for AH Development 

Published studies demonstrated a correlation between the presence and severity of NAFLD and the incidence of AH (Table 1). In the Korean population, hepatic steatosis detected by ultrasound imaging was an independent risk factor for AH [28]. This association persisted even after adjusting for multiple confounding factors, including changes in BMI (HR 1.36, 95% CI 1.10–1.67, *p* = 0.004) [29].

The fatty liver index (FLI), as a screening tool for steatosis, may also be an indirect marker of AH risk. Longitudinal studies demonstrated an increase in the risk of developing AH proportionately to the FLI increase, while FLI > 60 was considered a poor prognostic factor for the development of AH [32,35,36]. Even after adjusting for conventional risk factors and gender differences, the correlation persisted [36]. E. Siafi et al. (2023) discovered that FLI had an independent prognostic value for the development of cardiovascular events in hypertensive patients not receiving antihypertensive therapy [38]. Besides, J.H. Huh et al. (2015) confirmed that FLI was a predictor of the AH onset regardless of the presence of insulin resistance or systemic inflammation [31]. Since three of the four FLI variables (BMI, waist circumference, and serum triglyceride level) are criteria for metabolic syndrome, it is not clear whether the hepatic steatosis diagnosed by this index rather than metabolic disorders per se causes the development of AH. It is worth noting that the lack of direct measurement of intrahepatic fat content was the main limitation of the above studies, which must be considered when interpreting their results.

There is evidence that an increased content of liver fat (detected by magnetic resonance imaging) correlated with a higher risk of AH (HR 2.16, *p* = 0.025) [33]. Interestingly, with regression of steatosis, the risk of developing AH was comparable to that in the group of patients with a healthy liver [29,34]. However, patients with FLI < 60 had more consistent BP values within the normal range vs. patients with FLI ≥ 60 [38]. These findings suggest that improving liver health may reduce the risk of developing AH over time.

Fatty liver index was also investigated as a possible predictor for incident T2DM. For instance, D. Yadav et al. (2016) showed that, in a fully adjusted model that included baseline fasting blood glucose, baseline SBP, HDL, and homeostasis model assessment of insulin resistance (HOMA-IR), the ORs (95% CI) for new-onset T2DM in patients with FLI values of 30–59 and FLI values of ≥60 over 2.6 years were 1.87 (1.05–3.33) and 2.84 (1.40–5.75), respectively [39]. Similar results were published by I.H. Seo et al. (2022), who demonstrated that, after adjusting for potentially confounding factors, such as age, sex, waist circumference, alcohol intake, physical activity, mean arterial pressure, family history of diabetes, and HOMA-IR, the HRs of incident T2DM were 1.89 (95% CI 1.66–2.14) and 2.98 (95% CI 2.58–3.43) for the FLI values of 30–59 and of ≥60, respectively [40]. Moreover, E. García-Escobar et al. (2021) determined that the inclusion of FLI in the basic diabetes risk models (focused on the conventional risk factors for T2DM development, namely age, sex, fasting glucose level, and family history of T2DM in combination with HOMA-IR values or without them) allowed for correct reclassification of an additional 6.6% and 7% cases, respectively, in the overall study population [41]. In their study, D.J. Cuthbertson et al. (2021) clearly indicated that, apart from being a potential prognostic marker of NAFLD and T2DM, elevated FLI was also associated with the development of incident prediabetes in overweight and obese patients [42]. Possible mechanisms underlining the relationship between FLI and T2DM include hepatic insulin resistance and hepatic inflammation, as well as disturbed regulation of lipid and glucose metabolism in the settings of abnormal bile acid signaling [15,40].

### 3.2. AH as Independent Risk Factor for NAFLD Development 

While NAFLD can influence the development of AH, an inverse relationship is observed as well. There is increasing evidence that AH is a predictor of the development and progression of NAFLD (Table 2). It was established that, in the group of patients with AH, the prevalence of hepatic steatosis confirmed by the results of liver elastography was almost twice higher, while the risk of developing steatosis increased proportionately to the severity of AH [43]. In the Chinese cohort (*n* = 2049), a history of AH increased the risk of NAFLD nearly 1.5-fold [44]. In a Brazilian study (*n* = 5362), the HR was 1.8 [45]. However, timely control of AH with the achievement of target values (BP < 140/90 mmHg) reduced both the likelihood of developing NAFLD by over 40% and the likelihood of liver fibrosis progression [45]. 

In addition to predicting the onset of NAFLD, AH is also a risk factor for the progression of steatosis to steatohepatitis and advanced liver fibrosis [48]. It was demonstrated that, regardless of age, BMI, and the presence of diabetes mellitus, AH was an independent predictor of liver fibrosis based on noninvasive procedures (elastography and calculated indices: fibrosis-4 score [FIB-4], NAFLD fibrosis score [NFS], aspartate aminotransferase to platelet ratio index [APRI]) [44,49].

Besides, based on the analysis of serial liver biopsies, AH was established as an independent risk factor for predicting the progression of liver fibrosis over a mean follow-up period of 6.4 years [47]. The results of a meta-analysis based on seven published studies confirmed that the presence of AH at the time of the initial biopsy was associated with the development of progressive fibrosis in patients with NAFLD: odds ratio (OR) 1.94, 95% CI 1.00–3.74 [50]. 

### 3.3. Bidirectional Relationship 

Despite insufficient data to determine whether NAFLD is a consequence or a cause of AH, a strong bidirectional relationship between them has been confirmed by numerous studies [18,45].

Based on the results of the Dongfeng–Tongji cohort study, it can be concluded that the development and persistence of NAFLD over a 5-year follow-up period in patients without obesity and diabetes mellitus was associated with an increased risk of AH onset (OR 1.49 and 1.50, respectively). In fact, the development and persistence of AH predicted the onset of NAFLD (OR 1.45 and 1.61, correspondingly) [34]. Another prospective cohort study by J. Ma et al. (2017) with a 6-year follow-up period demonstrated a similar bidirectional relationship between signs of hepatic steatosis (according to computed tomography) and the risk of developing AH, which persisted even after adjusting for BMI and visceral adipose tissue volume [51]. The existence of a bidirectional association between NAFLD and AH regardless of common cardiometabolic risk factors was supported by the results of meta-analyses as well. A systematic review and meta-analysis of 390,348 patients detected an association between NAFLD and an increased risk of developing AH (HR 1.66, 95% CI 1.38–2.01, *p* < 0.001) [37]. Another meta-analysis (based on 11 studies) also revealed a strong bidirectional relationship. Thus, the presence of NAFLD was associated with a 1.55-fold increase in the risk of developing AH (95% CI 1.29–1.87; *n* = 46,487). On the other hand, the presence of AH was associated with a 1.63-fold increase in the risk of NAFLD (95% CI 1.41–1.88; *n* = 25,260) [52]. 

A significant correlation between AH and NAFLD emphasizes the need to identify risk groups for adverse outcomes, especially when such groups have anthropometric parameters within the norm. In this context, serum uric acid (SUA) level seems to be relevant. Indeed, in their study, J. He et al. (2022) demonstrated that SUA cut-off values of ≥478 µmol/L and ≥423.5 µmol/L were able to identify severe steatosis in men and women, respectively. Moreover, authors showed that higher SUA levels did predict severe steatosis even in lean patients with NAFLD [53]. Similar results were obtained in a 4-year prospective cohort study including 2832 subjects (33.97% had NAFLD at baseline). According to its results, elevated SUA levels may be used as an independent predictor of NAFLD [54]. Potential mechanisms mediating the relationship between NAFLD and elevated SUA level include insulin resistance, endothelial dysfunction, and systemic inflammation [55]. In addition, uric acid is able to induce oxidative stress, a decrease in endothelial nitric oxide availability, and an increase in both plasma renin activity and intrakidney angiotensin activity, thus leading to kidney vasoconstriction, ischemia, and salt-sensitive hypertension [56]. Elevated SUA levels are present in 25–60% of subjects with untreated essential hypertension, but also can serve as a prognostic factor for AH development [57,58]. It worth noting that there are several medications that are used “off label” and can be beneficial with regard to SUA level reduction, e.g., pioglitazone, losartan, and atorvastatin [55]. Of them, atorvastatin and losartan can be used for the treatment of both NAFLD and AH, thus emphasizing the possible commonality of the pathogenesis of these diseases and an important role of uric acid for their development and progression.

The other molecule that can unite two diseases is bilirubin. Serum bilirubin is a liver functional test that is widely used to assess liver function. It is important in the diagnosis and prognosis of patients with liver disorders, and it is included in all prognostic scores for liver diseases. In this context, the elevation of serum bilirubin is regarded as a marker of unfavorable outcomes. However, there is evidence indicating the association of a low level of bilirubin with increased risk of diseases, particularly cardiovascular disorders [59]. For instance, it was demonstrated that serum bilirubin level may be regarded as a marker for the assessment of left ventricular hypertrophy in patients with AH; hypertensive patients with left ventricular hypertrophy had lower bilirubin levels compared to those without left ventricular hypertrophy (*p* = 0.008) [60]. Similar results were obtained in the study by E.M. Bakirci et al. (2021), indicating that serum bilirubin levels may have a protective effect on the left atrium remodeling process in newly diagnosed hypertensive individuals [61].

At the same time, in the study by S.K. Kunutsor et al. (2017), it was shown that the hazard ratio for AH per 1-SD increase in total bilirubin level compared to baseline comprised 0.86 (95% CI, 0.81–0.92; *p* < 0.001). After adjustment for established risk factors (such as smoking status, alcohol consumption, history of diabetes mellitus, parental history of hypertension, body mass index, systolic blood pressure, total cholesterol, and estimated glomerular filtration rate) the hazard ratio was further attenuated to 0.94 (95% CI, 0.88–0.99; *p* = 0.040) [59]. In addition, there is evidence that elevated serum bilirubin may contribute to the development and progression of AH in newborns [62], as well as serve as a risk factor for death in patients with pulmonary artery hypertension [63]. Unfortunately, due to its bidirectional effect on the risk of cardiovascular disease, including AH, serum bilirubin does not appear to be suitable as a hypertension biomarker [64].

It is thought that protective effects of bilirubin in AH may be mainly attributed to its significant antioxidant properties, namely preventing vitamin A and polyunsaturated fatty acids from oxidation, as well as to its ability to scavenge hydrogen peroxide radicals [64]. Moreover, it was shown that bilirubin could reduce superoxide production via direct inhibition of NAD(P)H oxidases in the vasculature, which was associated with an increase in the bioavailability of nitric oxide [65]. According to the results of animal studies, elevated serum bilirubin level was protective against excessive vasoconstriction mediated by tubuloglomerular feedback and was able to attenuate vasoconstriction in response to high levels of vasoconstrictors such as angiotensin II [66]. The other possible mechanisms of the antihypertensive action of bilirubin include regulation of the endothelin production and signaling (via influence on the preproendothelin gene transcription), as well as the improvement in calcium handling in vascular smooth muscle cells [67].

Although both conjugated and unconjugated bilirubin display antioxidant effects, there is relatively little evidence in favor of the potential beneficial effects of conjugated bilirubin in vivo for the following reasons: (1) serum conjugated bilirubin concentrations remain markedly below those of unconjugated bilirubin, and (2) conjugated bilirubin in general increases in the course of hepatobiliary disease, which may hamper the evaluation of any possible protective effects [68].

It is alarming that the combination of AH and NAFLD can remain undetected for a long time, and the rate of disease progression in this case varies from one patient to another and depends on multiple pathogenetic factors. In the literature, the term “silent killer” is used for each of these diseases since they have a long, asymptomatic course that causes delays in diagnosis and treatment which can lead to serious complications and death [69].

## 4. Mechanisms of the Relationship between NAFLD and AH

**Figure 1:**
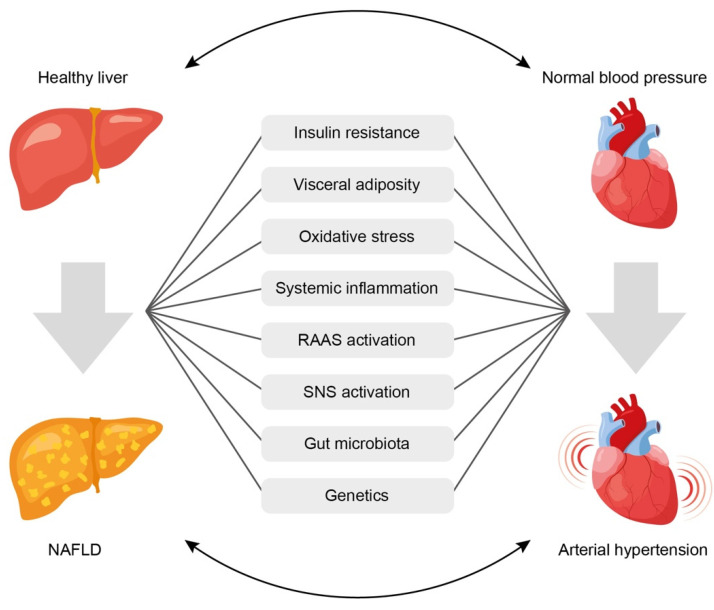
Common pathophysiological links underlining the association between arterial hypertension and non-alcoholic fatty liver disease. See text for explanations. NAFLD = non-alcoholic fatty liver disease; RAAS = renin-angiotensin-aldosterone system; SNS = sympathetic nervous system.

Despite the presence of bidirectional relationship between NAFLD and AH, it is often difficult to establish which of the two conditions occurs first. However, the existence of common mechanisms determines the high risk of developing one of them in cases where the other is already present. The mechanisms of the relationship between NAFLD and AH are mostly known (Figure 1) [70], but they continue to be explored in more depth. Common links in pathogenesis that may explain the potential pathophysiological relationship between NAFLD and AH include:

(1) Insulin resistance,
(2) Systemic inflammation,
(3) Activation of the renin-angiotensin-aldosterone system (RAAS),
(4) Oxidative stress,
(5) Activation of the sympathetic nervous system,
(6) Gut microbiota disbalance,
(7) Genetic factors.

### 4.1. Insulin Resistance

Uncontrolled AH leads to a decrease in peripheral circulation, which contributes to a reduction in the sensitivity of peripheral tissues to insulin and, consequently, to hyperinsulinemia. The latter stimulates the proliferation of smooth muscle cells and vascular fibroblasts, which leads to a narrowing of their lumen and an increase in total peripheral vascular resistance (TPVR). These vascular effects of insulin are mediated through the mitogen-activated protein kinase (MAPK) pathway, which promotes secretion of the vasoconstrictor endothelin-1 (ET-1). The other pathway that may be involved in the vascular deleterious effects of insulin is the phosphatidylinositol 3-kinase (PI3K)-dependent insulin signaling required for metabolic actions of insulin. It was shown that the impairment of PI3K/protein kinase B (Akt) pathway consistent with insulin resistance caused by glucotoxicity, lipotoxicity, or inflammation predicted diminished NO production and increased ET-1 secretion characteristic of diabetes and endothelial dysfunction [71]. Moreover, according to experimental data, hyperinsulinemia increases the activity of the central sections of the sympathetic nervous system, accompanied by augmented sympathetic stimulation of the heart, blood vessels, and kidneys [72].

There is evidence that compensatory hyperinsulinemia contributes to the activation of the RAAS and the melanocortin system of the brain, which play a pivotal role in the onset of AH [73]. Upon activation of the RAAS, a cascade of reactions occurs, including the production of angiotensin II, which causes spasm of smooth muscles of arterioles, an increase in hydrostatic pressure in the glomeruli, along with activation of aldosterone synthesis and higher sodium reabsorption in the kidneys. This ultimately leads to an increase in circulating blood volume and elevated BP. The kidneys are also a target organ for hyperinsulinemia. Insulin receptors are expressed on renal tubular cells and podocytes. Under physiological conditions, insulin induces vasodilation by increasing endothelial nitric oxide production through activation of the PI3K/ Akt pathway. In insulin resistance, this pathway is disrupted, and insulin triggers the MAPK pathway, which promotes renal vasoconstriction [18,72]. Besides this, under conditions of hyperinsulinemia, the activity of transmembrane ion-exchange Na^+^, K^+^ and Ca^2+^ ATPases is disrupted, while sodium reabsorption in the nephron tubules increases, which leads to fluid retention and the development of hypervolemia, as well as an increase in the sodium content in the blood vessel walls and their spasm [72].

### 4.2. Visceral Obesity

Visceral adipose tissue is an independent endocrine organ. Adipocytes secrete hormonally active molecules called adipokines (tumor necrosis factor α [TNF-α], IL-6, leptin, etc.), which enhance the pathological effects of insulin. TNF-α and IL-6 trigger the processes of cytotoxic inflammatory responses and chronic persistent inflammation both in the liver and in the vascular walls [74]. Adipocytes are also capable of synthesizing angiotensin II, thereby regulating BP levels [75].

It is known that adipokines can influence the RAAS. An increase in leptin levels against the background of a high-fat diet in rats caused the activation of the RAAS and the production of proinflammatory cytokines in the brain, which led to a progressive increase in BP [76]. Accordingly, L. D’Elia et al. (2021) showed that leptin values greater than 2.9 ng/mL were associated with a twofold increased risk of developing arterial stiffening in a sample of adult men without baseline arterial stiffening and antihypertensive treatment at baseline during an 8-year follow-up. These results were independent of body weight and BP [77]. Altered adipokine profile characterized by an increase in leptin concentration and a decrease in adiponectin levels was also described in NAFLD, thus indicating an additional common pathophysiological link between NAFLD and AH [78]. In addition to the effects described at the beginning of this paragraph, unfavorable action of leptin on BP level may be also attributed to the following mechanisms: reduction in NO bioavailability and regulation of ET-1 expression, as well as stimulation of endothelial cell growth and cardiovascular smooth muscle cell proliferation, increase in sympathetic nerve activity, and impairment in sodium handling [79,80].

An excess of free fatty acids (FFAs) released by adipose tissue under conditions of insulin resistance contributes to the formation of ectopic fat deposits in the form of perivascular and perirenal fat depots. In the insulin-resistant state, perivascular fat cells increase in size and number, and secrete reduced levels of regenerative factors, such as hepatocyte growth factor, and increased levels of proinflammatory factors, such as IL-6, TNF-α and monocyte chemoattractive factor 1 MCP-1. These proinflammatory factors inhibit the PI3K/Akt axis of insulin signal transduction, increase endothelial permeability and enhance insulin resistance [72]. This promotes the remodeling of the vascular wall with an increase in arterial stiffness and a resulting increase in pulse pressure [72,75,81]. Perirenal fat, along with inflicting mechanical pressure, is lipotoxic and can impair renal function in a paracrine manner, which results in RAAS activation and increased sodium reabsorption [75].

### 4.3. Oxidative Stress and Biologically Active Substances

Excessive release of FFAs from adipose tissue is lipotoxic to the liver. Against the background of mitochondrial dysfunction, the processes of lipid peroxidation are triggered with the formation of free radicals. An increase in free radical activity is the major process leading to the release of proinflammatory profibrogenic cytokines and hepatokines (fetuin-A, fibroblast growth factor 21 [FGF-21], and selenoprotein P) [82].

Some authors revealed the relationship between NAFLD and the biomarker of systemic endothelial dysfunction, E-selectin. E-selectin is a cell adhesion molecule expressed on endothelial cells; it is produced when they are damaged by cytokines. It was shown that the extent of NAFLD histological activity correlated with the level of E-selectin expression in the liver and the concentration of E-selectin in blood plasma [83]. Also, it was established that patients with AH and NAFLD had more pronounced endothelial function disorders, according to the values of arterial stiffness, compared with patients with isolated AH [84]. NAFLD is characterized by a change in the profile of hepatokines, which are biologically active substances secreted by hepatocytes. It was confirmed that they can participate in the development of systemic insulin resistance both directly (by influencing the insulin signaling pathway) and indirectly (by regulating lipid and glucose metabolism) [82]. For instance, in the study by T.W. Jung et al. (2013), it was demonstrated that fetuin-A might directly cause insulin resistance and modulate inflammatory reactions via stimulation of triacylglycerol accumulation in hepatocytes [85]. Accordingly, a significant decrease in circulating fetuin-A levels after 12 weeks of caloric restriction was accompanied by improvements in visceral fat area, blood pressure, lipid profiles, and glucose levels [86].

Regarding FGF-21, it was shown that circulating FGF-21 positively correlated with the brachial–ankle pulse wave velocity reflecting arterial stiffness. This suggests that FGF-21 might also be secreted by endothelial cells (not only hepatocytes) in response to stress, and that its elevated levels may be a signal of endothelial cell injury [87].

Regarding selenoprotein P, most published studies focus on the investigation of its role in the development and progression of pulmonary hypertension. Its effects in this condition are explained by the promotion of cell proliferation and apoptosis resistance through increased oxidative stress and mitochondrial dysfunction, which in turn are associated with activated hypoxia-inducible factor-1α and dysregulated glutathione metabolism [88,89].

The development of oxidative stress and the presence of systemic inflammation, accompanied by the circulation of TNF-α, IL-6, and advanced glycation end products, are key components of endothelial dysfunction. They can lead to both micro- and macroangiopathy and can contribute to progressive pathological remodeling of the vascular wall [81,90,91]. Elevated production of proinflammatory cytokines is a crucial mechanism involved in the progression of AH. TNF-α and IL-6 can regulate the expression of RAAS components, especially the production of angiotensinogen, which causes vasoconstriction, affects renal hemodynamics, and ultimately leads to an increased BP [92].

The presence of hepatic steatosis can negatively affect the regulation of the cardiovascular function via the autonomic nervous system. In patients with NAFLD, autonomic dysfunction was revealed in the form of an increased sympathetic activity and an impaired response to parasympathetic signals. Accordingly, the heart rate increased, and cardiac output decreased, which caused an additional impact on the cardiovascular system [93].

### 4.4. Gut Microbiota

An altered composition of the intestinal microbiota and a change in the permeability of the intestinal mucosa play an important role in the pathogenesis of both NAFLD and AH. The mechanisms of the gut microbiota impact on the development and progression of NAFLD and AH are caused by complex interactions between the gut microbiota and the host organism. Likely factors include metabolism of choline, bile acids, and amino acids resulting in synthesis of vasoactive hormones, such as trimethylamine (TMA) and trimethylamine N-oxide (TMAO), uremic toxins (indoxyl sulfate and p-cresol sulfate), as well as production of short-chain fatty acids (SCFAs) and ethanol by intestinal microorganisms [94,95]. Next, we discuss the role of SCFAs, TMA, and intestinal permeability in the pathogenesis of both AH and NAFLD.

#### 4.4.1. Short-Chain Fatty Acids

In patients with AH and NAFLD, a reduction in the bacterial diversity of the intestinal microbiota and a decrease in the production of SCFA were revealed. The most important and biologically active SCFAs include acetate, propionate, and butyrate; they are made from dietary fiber in the intestines. Regarding their role in the pathogenesis of AH, animal studies suggested that SCFAs may have both hypotensive and hypertensive effects depending on the receptors they bind to [95,96]. For instance, propionate-induced activation of the G protein-coupled receptor (GPR) 42 in vascular endothelium caused a decrease in BP in Olfr78−/− mice [97]. In contrast, binding of propionate or acetate to the olfactory receptor (Olfr 78 in mice and OR51E2 in humans) increased BP, possibly due to their effect on vascular smooth muscle cells in renal afferent arterioles and peripheral blood vessels, as well as renin production [98]. The opposite effect of SCFAs on BP can be explained by the different sensitivity of Olfr78 and GPR41 to SCFAs. Indeed, GPR41 receptors are activated by basal serum concentrations of SCFAs ranging from 0.1 to 0.9 mmol, resulting in vasodilation and a reduction in BP. However, only higher serum levels of SCFA can activate Olfr78, thereby increasing renin production and BP values [98]. 

Moreover, by acting on GPR expressed in the sympathetic ganglia, SCFAs can directly regulate the sympathetic nervous system. They can also activate vagal afferent neurons and directly affect the function of the central nervous system. Such mechanisms of SCFA action on the central and peripheral nervous system lead to a decrease in BP [99].

The other mechanism of BP regulation by SCFAs includes their impact on the immune pathways, including intestinal and immune homeostasis, inflammatory cell biology and inflammatory response [99]. For instance, it was shown that butyrate was able to decrease the IL-6 and TNF-α levels caused by angiotensin II in vitro and induce regulatory T cells differentiation in vivo and in vitro. In addition, this SCFA could reduce IL-17 levels in patients with hypertension [100,101]. The anti-inflammatory effect of SCFAs, especially butyrate, may be also mediated by histone deacetylase inhibition in vascular endothelial cells, thereby contributing to the prevention of vascular inflammation [102].

Potential mechanisms of protective action of SCFAs on the liver include reduction of fat accumulation by promoting lipolysis, fatty acid oxidation, inhibition of fatty acid synthesis [103,104], maintenance of intestinal barrier function [105], regulation of intestinal motility [106], and suppression of inflammation and steatosis in the liver [107,108].

Since both NAFLD and AH are believed to be associated with metabolic disorders, the described protective effects of SCFAs, which are important for the pathogenesis of NAFLD, may also play a role in the development and progression of AH.

#### 4.4.2. Trimethylamine and Trimethylamine N-oxide 

Despite many studies examining the role of TMA and TMAO in the development and progression of NAFLD, there is no consensus regarding their effects on this liver disease. Some studies suggest that TMAO contributes to liver damage and disease progression [109,110,111,112], while others argue that TMA and TMAO may have a protective effect via improving lipid metabolism disorders, endoplasmic reticulum stress, and reducing cell death under lipid overload conditions [113,114,115]. The presented data suggest that the effect of TMAO may vary and strongly depends on the levels of other substrates in plasma, such as cholesterol [116]. Similarly, the role of TMA/TMAO in the development of AH remains uncertain. Some published data imply a vasoconstrictive mechanism of action of TMA/TMAO at high doses [117,118], while at low concentrations, these substances are thought to reduce diastolic dysfunction and cardiac fibrosis [119]. These data were confirmed by the results of meta-analysis published by X. Ge et al. (2020), indicating that, in comparison with low circulating TMAO concentrations, high TMAO concentrations were associated with a higher prevalence of hypertension (risk ratio [RR]: 1.12; 95% CI: 1.06, 1.17; *p* < 0.0001) [120]. 

Several potential mechanisms by which TMAO promotes hypertension include [121]: (1) enhanced angiotensin II-induced vasoconstriction and acute pressor response. This mechanism is implemented through the activation of the protein kinase RNA-like endoplasmic reticulum kinase (PERK) pathway, leading to apoptosis, inflammation, and vascular injury [122,123]; (2) the upregulation of scavenger receptors on the surface of macrophages and the stimulation of foam cell formation, atherosclerosis, vascular constriction and arterial stiffening [122,123]; (3) the disturbance of the reverse transport of cholesterol from extrahepatic organs and tissues into the liver resulting in increased oxidized-low density lipoprotein deposition in peripheral tissues, which contributes to atherosclerosis progression and increases the risk of CVD [124]; (4) increased production of proinflammatory cytokines, such as IL-1β, IL-18, TNF-α in combination with decreased production of anti-inflammatory cytokines, in particular IL-10 [123,125]; (5) participation in the development of renal dysfunction [126], and (6) the induction of cardiac dysfunction at high serum concentrations [127] by facilitating cardiac mitochondrial dysfunction, myocardial hypertrophy, and fibrosis [128].

#### 4.4.3. Increased Intestinal Permeability

With increased intestinal permeability, gram-negative bacterial lipopolysaccharides reach the portal circulation and stimulate the development of systemic inflammation through toll-like receptors [129,130,131]. Moreover, it was shown that high circulating endotoxin levels resulted in the release of pro-inflammatory cytokines, including IL-1β, IL-12, IL-6, and TNF-α [132]. At the same time, high circulating levels of pro-inflammatory cytokines may contribute to intestinal junction disassembly and increase in gut permeability [133]. Indeed, an increase in BP was associated with a decrease in the level of tight junction proteins (occludin, tight junction protein 1 and claudin 4) in the rat model of AH compared with the control group [134]. The negative impact of arterial hypertension on gut barrier permeability may be explained by several possible mechanisms. The first one is the decreased intestinal blood flow due to changes in arterioles, particularly, thickening of the walls and narrowing of the lumens. This may lead to intestinal mucosa damage and barrier impairment. The second mechanism involves gut microbiota dysbiosis characterized by decreased microbial richness and diversity, reduced SCFAs production, and overgrowth of opportunistic pathogens [135].

According to the results of a meta-analysis conducted by J. Luther et al. (2015), almost 39.1% of NAFLD patients included in the analysis (*n* = 128) had signs of increased intestinal permeability vs. 6.8% in the control group of healthy individuals [136]. These data were further supported by another meta-analysis showing that impaired intestinal permeability in patients with NAFLD was associated with the grade of hepatic steatosis [137]. Similarly to patients with AH, it was assumed that patients with NAFLD (and especially with cirrhosis associated with NASH) had qualitative and quantitative changes in tight junction proteins [138,139]. Increased translocation of bacterial products was believed to lead to inflammation and fibrogenesis in the liver through toll-like receptor 4 stimulation. However, the relationship between intestinal permeability and fibrosis stage in NAFLD has not yet been established [140].

### 4.5. Genetic Factors

In addition to external factors, the role of genetic factors in the development of NAFLD and AH have been investigated. In the study of overlapping genes, 13 common genes for NAFLD and AH were identified. Besides this, four more common genes have been identified: leptin (LEP), adiponectin (ADIPOQ), aryl hydrocarbon receptor (AHR), and transforming growth factor beta-1 (TGFB1) genes, the expression of which is typical for patients with AH, NAFLD, liver fibrosis, and the presence of systemic inflammation [141]. In addition, C. Ma et al. (2021) showed that hypertension genes were more adjacent to NAFLD genes than random genes in the protein–protein interaction network, indicating a strong association between these two diseases [141].

According to expression information obtained from Gene cards database, both RNA-sequencing and microarray data indicated that RAS constituents, including a classical angiotensin-converting enzyme (ACE)/angiotensin II/type 1 angiotensin receptor axis and an alternative angiotensin-converting enzyme 2/angiotensin 1–7/Mas axis, were expressed in normal liver, heart and kidney. It is well-established that angiotensin II promotes insulin resistance, de novo lipogenesis, and pro-inflammatory cytokine production, and triggers liver inflammation and fibrogenesis [142], while active angiotensin 1–7 signal inhibits liver lipogenesis, fatty acid oxidation, inflammation, and fibrosis [143].

Another gene that seems to be related to both liver physiology and hypertension-NAFLD interaction is the aldehyde dehydrogenase (ALDH1A1, alternatively known as retinaldehyde dehydrogenase 1 or RALDH1) gene, encoding the enzyme which catalyzes the second and irreversible step of retinaldehyde oxidation to vitamin A (retinoic acid) [141]. According to the study by G. Zhong et al. (2019), the expression of ALDH1A1 was significantly higher in the livers of NASH patients than in the livers of healthy volunteers [144]. Moreover, analysis of the Oncomine database demonstrated that the expression of ALDH1A1 was significantly upregulated in the hepatocellular carcinoma tissues than in the normal tissues [145]. It is considered that ALDH1A1 might play an important role in the detoxification of lipid-derived aldehydes, including 4-hydroxy-2-nonenal and acrolein, which are known for their ability to mediate oxidative stress [146]. Additionally, through binding to either retinoic acid receptors (RARs) or retinoid X receptors (RXRs), retinoic acid may exert opposite effects on lipid metabolism in the liver: RARs binding leads to the suppression of hepatic non-esterified free fatty acids and triglyceride accumulation, while RXR-mediated signaling may cause hepatic lipid accumulation [147].

It was also revealed that polymorphism of the angiotensin II receptor type 1 (AGTR1) gene was indicative of the occurrence of NAFLD and AH in patients without obesity, insulin resistance, or hyperlipidemia. Some studies demonstrated that carriers of the C allele of the AGTR1 gene exhibited an elevated release of FFAs from adipose tissue and an imbalance of adipokines with a predominance of proinflammatory adipokines and chemokines over anti-inflammatory adiponectin [148].

Understanding the shared genes and biological mechanisms of AH and NAFLD can help to develop combined preventive strategies, explore novel therapeutic approaches against NAFLD, and design the most suitable treatment plan for patients with comorbidity of AH and NAFLD.

### 4.6. Influence of Gender on the Development of AH and NAFLD

It is well known that the frequency and prevalence of AH and NAFLD depend on the gender and age of the patient.

Most current guidelines for the management of AH patients agree that its prevalence among men and women of different age groups is different [149,150]. This may be due to various biological and physiological factors, as well as their interaction [151]. Similar to NAFLD, the prevalence of AH in the age group under 50 years is significantly higher in men than in women, while after reaching 50 years of age, the prevalence of AH in women is approximately the same as it is in men, or even slightly higher [149,150,152]. The likely biological factors determining the unequal prevalence of AH in men and women are the effects of sex hormones and differences in the sex chromosomes [153], while probable behavioral factors are high values of body mass index [154], smoking [155] and low physical activity [156]. 

A twelve-year Japanese study established that the prevalence of fatty liver in men averaged 26% vs. 13% in women. Also, it was shown that the frequency of NAFLD in women increases with age to a greater extent than in men, and the maximum differences were recorded in the age group of 70–79 years. On the contrary, the incidence of this pathology in men was similar in all age groups [157,158,159]. Another study from South China reported that the prevalence of NAFLD in the age group under 50 years old was significantly higher in men compared with women (22.4% vs. 7.1%, respectively, *p* < 0.001). However, this pattern was reversed in the age group of 50 years and older (20.6% vs. 27.6%, respectively, *p* < 0.05) [158,160].

There is strong evidence suggesting an important role of sex hormones in the onset of NAFLD. A meta-analysis showed that an increase in serum testosterone levels in women was also observed in polycystic ovary syndrome (PCOS), which is one of the conditions associated with NAFLD; this was accompanied by an increased risk of developing NAFLD in women. Hypogonadism associated with PCOS was shown to double the risk of developing NAFLD and contribute to an increase in obesity, insulin resistance, and metabolic syndrome [161,162,163]. Meanwhile, a decrease in serum testosterone levels is associated with the risk of hepatic steatosis in men [164]. However, a few studies suggested some protective effect of androgens regarding the development of hepatic steatosis. They can promote exocytosis of very-low-density lipoproteins (VLDL), inhibition of de novo lipogenesis, maintenance of carbohydrate metabolism, and fatty acid beta-oxidation [165,166].

Estrogens play a protective role against the development of hepatic steatosis in both men and women. This effect is implemented due to the ability of estrogens to increase insulin sensitivity, reduce the synthesis of triglycerides, and activate free fatty acid oxidation in the liver, as well as improve mitochondrial function and reduce the severity of inflammation in the liver tissue [165,167,168,169]. Estrogens are also involved in maintaining cholesterol metabolism in the liver by means of ensuring the synthesis of lipoproteins, secretion of VLDL, increased formation of high-density lipoproteins (HDL) and elimination of oxidized low-density lipoproteins (LDL). 

In addition to the well-known role of sex hormones in the development of metabolic syndrome and related disorders, some studies elucidated that sex chromosomes per se may be responsible for the sexual dimorphism seen in NAFLD and metabolic syndrome. Sex chromosomes independent of gonadal status in a mouse model were associated with simple steatosis and NAFLD [170]. A study by Chen et al. (2016) demonstrated that the sex chromosome complement, XX or XY, affected adiposity and weight gain, because XX mice exhibited higher adiposity than XY mice even after a gonadectomy was performed to eliminate the effect of sex hormones [171]. It has been argued that increased adiposity was due to the presence of an extra X chromosome rather than the absence of a Y chromosome, as shown by genetic studies in mice with XO and XXY chromosomes. The established mechanism involved certain genes avoiding X chromosome inactivation and exhibiting increased levels of expression in the adipose tissue and liver of XX mice rather than XY mice [158,171]. 

With regard to the combination of NAFLD and AH, the following age- and gender-related characteristics have been described. First, women with NAFLD had a higher prevalence of essential hypertension (75.8%) [172]. Second, women with AH were more likely to develop NAFLD within 5 years [34]. Female gender is associated with a greater likelihood of NAFLD progression with the development of advanced liver fibrosis [173], especially at the age of over 50 years, which can lead to the development of pathogenetic mechanisms of AH, viz., an increase in arterial wall stiffness and endothelial dysfunction [174].

Gender-based differences in the prevalence of AH appear multifactorial as well, and their causes are not fully understood. In recent years, differences in the activity of the sympathetic nervous system and arterial wall stiffness were widely discussed, in the genesis of which sex hormones may play a certain role [175]. It should be noted that dysfunction of the autonomic nervous system may be more important for the development of AH in women than in men [176]. Furthermore, the age-related increase in autonomic nervous system activity is more pronounced in women than in men and does not depend on body mass index or menopause [177,178]. It is well known that the risk and incidence of AH in premenopausal women is lower than in men, but after 65 years of age, the prevalence of AH becomes higher in women [179].

Androgens and estrogens are able to regulate BP by influencing the RAAS. Androgens stimulate the RAAS, which leads to an elevated BP [180], while ovarian hormones have the opposite effect via reducing the activity of renin and ACE in blood plasma [181]. The effect of sex hormones on renal sodium reabsorption and vascular resistance may also explain differences between men and women in BP control [180,182]. Estrogens are likely to have the ability to maintain normal endothelial function by stimulating nitric oxide (NO) production including positive effects on arterial wall structure and function, which, in turn, help reduce vascular wall stiffness. Moreover, they reduce the effects of the sympathetic nervous system [179,183].

## 5. Conclusions

Accumulated evidence suggests a significant bidirectional relationship between hypertension and non-alcoholic fatty liver disease, which can be independent of other metabolic syndrome components and may be explained by the existence of pathophysiological mechanisms that are common for both diseases. Considering the poorer prognosis in the comorbidity of AH and NAFLD demonstrated in clinical studies, active detection of AH in patients with NAFLD, as well as screening patients with AH for NAFLD, seems crucial. Considering AH as a predictive marker of NAFLD, and vice versa, may help predict risks of CVD development and mortality in these patient groups. Timely detection and treatment of AH can serve as a potentially important aspect of preventing the development and progression of NAFLD. 

## Figures and Tables

**Table 1:** NAFLD as an independent factor of arterial hypertension.

Reference	Study Design	Study Population	NAFLD Diagnosis	Follow-Up	Key Results
R.L. Vasunta et al., 2012 [30]	Analysis of the dataset from the population-based epidemiological case–control OPERA (The Oulu Project Elucidating Risk of Atherosclerosis) study. Cross-sectional design	890 hypertensive (*n* = 433) and normotensive (*n* = 457) individuals (mean age ± SD 51.0 ± 6.0 years; 49% were males)	Abdominal ultrasound	N/A	After adjusting for BMI, gender and age, individuals with NAFLD had significantly higher ambulatory daytime (*p* < 0.02) and nighttime (*p* < 0.05) SBP values, compared with individuals without fatty liver disease
J.H. Ryoo et al., 2014 [28]	Prospective cohort study	22,090 Korean men without AH (mean age ± SD: 42.1 ± 6.8 years). NAFLD was diagnosed in 7561 patients (34.2%)	Abdominal ultrasound, exclusion of liver disease of other etiology	5 years	The incidence of AH was higher in patients with NAFLD vs. those without it (30.1% vs. 14.4%, *p* < 0.001). After adjusting for multiple covariates, the OR for developing AH were higher in mild NAFLD patients (1.07; 95% CI 1.00–1.15) and moderate to severe NAFLD patients (1.14; 95% CI 1.00–1.30), compared with the control group (*p* < 0.001)
K.C. Sung et al., 2014 [29]	Retrospective cohort study	11,448 patients without AH at baseline. Of them, 911 patients developed AH (mean age ± SD: 42.28 ± 6.45 years; 84.4% were males), and 1418 patients developed fatty liver (mean age ± SD: 40.84 ± 5.58 years; 83.9% were males) during follow-up	Abdominal ultrasound, exclusion of liver disease of other etiology	5 years	The development of fatty liver was associated with the incidence of AH even after adjusting for multiple confounding factors (adjusted OR 1.60; 95% CI 1.30, 1.96; *p* < 0.001).
J.H. Huh et al., 2015 [31]	Prospective cohort study (analysis of the data from the Korean Genome and Epidemiology Study on Atherosclerosis Risk of Rural Areas in the Korean General Population [KoGES-ARIRANG])	1521 adults (31.8% were males) aged 40 to 70 years without AH at baseline	FLI	Mean follow-up period was 2.6 years	During the follow-up period, 153 subjects (10.06%) developed AH. The OR values (95% CI) for developing AH in the 30 ≤ FLI ≤ 59 group and FLI ≥ 60 group were 1.87 (1.2–2.91) and 2.22 (1.16–4.25), respectively, compared with values in the FLI < 30 group, after adjustment for confounding factors (e.g., liver enzyme activity, adiponectin, IR, and etc.)
F. Bonnet et al., 2017 [32]	Analysis of data from two longitudinal studies in subjects without AH: D.E.S.I.R. cohort (large 9-year prospective cohort epidemiological study on the insulin resistance syndrome) RISC (Relationship between insulin Sensitivity and Cardiovascular disease) cohort (3-year prospective study)	Men and women aged 30–65 years; *n* = 2565 (D.E.S.I.R. cohort) and *n* = 321 (RISC cohort). Of them, 1021 subjects (39.8%) from the D.E.S.I.R. cohort (mean age ± SD: 48 ± 9 years; 54% were males) and 63 (19.6%) subjects from the RISC cohort (mean age ± SD: 47.6 ± 7.7 years; 62% were males) developed AH during follow-up	FLI	9 years 3 years	After adjusting for confounding factors, only GGT was significantly associated with the onset of AH (standardized OR, 1.21; 95% CI, 1.10–1.34; *p* = 0.0001). FLI as a continuous value or ≥60 at baseline was predictive of the onset incidence of AH in the multivariate model
R. Lorbeer et al., 2017 [33]	Cross-sectional data from the MRI subset of the KORA FF4 study	384 participants (58.1% males) aged 39–73 years	HFF measured in the left and right lobe of the liver using single voxel multi-echo ^1^H-spectroscopy and at the level of the portal vein using a multi-echo Dixon-sequence (MRI)	N/A	The best prediction of AH among all HFF variables was observed for the left lobe HFF threshold (3.57%; OR = 2.62, *p* = 0.003). Alcohol consumption appeared to be an effect modifier for the association between HFF and AH (non-drinkers: OR = 3.76, *p* = 0.025; alcohol consumers: OR = 1.59, *p* = 0.165).
P. Liu et al., 2018 [34]	Analysis of the dataset from the Dongfeng–Tongji cohort study	6704 eligible hypertension-free subjects and 9328 NAFLD-free subjects at baseline (mean age 59.8 ± 7.6 years; 36.3% were males)	Abdominal ultrasound, exclusion of liver disease of other etiology	N/A	Incidental NAFLD, as well as persistent NAFLD, were significantly associated with an increased OR for incidental AH (OR 1.49, 95% CI 1.26–1.76, *p* < 0.0001 and OR 1.50, 95% CI 1.27–1.78, *p* < 0.0001, respectively). At the same time, incidental AH was associated with risk of incidental NAFLD (OR 1.45, 95% CI 1.23–1.71, *p* < 0.0001). Similar data were obtained for persistent AH (OR 1.61, 95% CI 1.35–1.92, *p* < 0.0001)
J.H. Roh et al., 2020 [35]	Retrospective analysis of the data set from the National Health Insurance Service–National Sample Cohort 2.0 (NHIS-NSC 2.0)	334,280 healthy Korean people with no known comorbidities aged ≥20 years. Of these, 24,678 subjects (7.4%) developed AH during follow-up (60.6% aged ≥ 50 years, 51.1% men)	FLI The following quartiles of the FLI values were suggested: Q1, 0–4.9; Q2, 5.0–12.5; Q3, 12.6–31.0; Q4, >31.0.	The median follow-up was 5.2 years (interquartile range: 3.5–6.3)	The highest FLI values were associated with an increased risk of new onset AH (adjusted OR between Q4 and Q1 FLI values 2.330; 95% CI 2.218–2.448; *p* < 0.001).
Y. Higashiura et al., 2021 [36]	Retrospective cohort study	15,965 subjects (9566 males, mean age ± SD: 45 ± 11 years; 6499 females, mean age ± SD 45 ± 11 years)	FLI	The mean follow-up period was 6.0 years (range: 1–10 years)	Over a 10-year period, 2,304 men (24.3%) and 745 women (11.5%) developed AH. The combination of FLI with traditional risk factors significantly improved the discriminatory power of the AH risk stratification model for both men and women (*p* < 0.001)
S. Ciardullo et al., 2022 [37]	A systematic review and meta-analysis of 11 observational studies	390,348 middle-aged individuals (52% men)	Abdominal ultrasound in 6 studies *(n* = 45,924) Computed tomography in 1 study (*n* = 1051) FLI in 4 studies (*n* = 343,373)	Mean follow-up period was 5.7 years (range, 2.6–9 years)	NAFLD was associated with a 1.6-fold increase in the risk of developing AH
E. Siafi et al., 2023 [38]	Prospective cohort study	903 hypertensive patients without a history of cardiovascular disease (mean age ± SD: 52.7 ± 11.4 years; 55% were males)	FLI	Mean follow-up period was 5.2 ± 3.2 years	Patients with FLI < 60 (*n* = 625) had better BP control vs. their counterparts with FLI ≥ 60 (*n* = 278) at follow-up (43% vs 33%, *p* = 0.02).

AH = arterial hypertension; BMI = body mass index; BP = blood pressure; CI = confidence interval; DBP = diastolic blood pressure; FLI = fatty liver index; HFF = hepatic fat fraction; hsCRP = high-sensitivity C-reactive protein; IR = insulin resistance; MRI = magnetic resonance imagining; N/A = not applicable; NAFLD = non-alcoholic fatty liver disease; OR = odds ratio; SBP = systolic blood pressure; SD = standard deviation.

**Table 2:** Arterial hypertension as an independent risk factor for NAFLD.

Reference	Study Design	Study Population	NAFLD Diagnosis	Follow-Up	Key Results
A. Tsuneto et al., 2010 [46]	Prospective cohort study	1635 Nagasaki atomic bomb survivors (606 men) without fatty liver at baseline (mean age ± SD: 63.1 ± 8.9 years; 37.1% were males)	Abdominal ultrasound, exclusion of liver disease of other etiology	The mean follow-up duration was 11.6 years (SD, 4.6; median, 14.0; range, 1.3–17.1)	AH was an independent predictor of NAFLD (relative risk 1.63; 95%CI, 1.30–2.04; *p* < 0.001 after adjusting for age, gender, smoking and drinking habits; risk ratio 1.31; 95% CI, 1.01–1.71; *p* = 0.046)
P. Sorrentino et al., 2010 [47]	Prospective cohort study	271 obese patients with NAFLD and abnormal liver enzymes. 132 patients were included in the final analysis (mean age ± SD: 41.5 ± 9.2 years; 40.15% were males)	Liver biopsy	The median follow-up was 6.4 years (range: 5–8.3 years).	The presence of AH at baseline was independently associated with aggravation of hepatic fibrosis during the follow-up (OR 4.8; 95% CI, 2.7–18.2; *p* = 0.028)
E.C. Aneni et al., 2015 [45]	Cross-sectional study	A Brazilian cohort of 5362 healthy middle-aged men and women (without a history of cardiovascular or liver disease) Mean age ± SD: 43.5 ± 9.3 years; 77% were males)	Abdominal ultrasound, exclusion of liver disease of other etiology, FIB-4	N/A	Multivariate analysis demonstrated that AH was associated with increased risk of NAFLD (adjusted OR 1.8; 95% CI 1.4–2.3).
Y. Wang et al., 2016 [43]	Retrospective cohort study	836 subjects (mean age ± SD: 53.6 ± 13.5 years), of them, 333 (39.83%) had NAFLD	Hepatic transient elastography with the assessment of controlled attenuation parameter (CAP). CAP cut-off was ≥ 238 dB/m. exclusion of the secondary causes of hepatic steatosis.	N/A	In patients with AH, NAFLD was independently associated with AH and BP category (OR for NAFLD was 1.476; 95% CI 1.166–2.551 when comparing patients with SBP ≥ 180 mmHg and/or DBP ≥ 110 mmHg and patients with normal BP)
Q. Huang et al., 2023 [44]	Meta-analysis (11 cross-sectional studies)	2049 adults aged ≥20 years (42.5% were males). Of them, 804 (39.2%) had NAFLD at baseline (mean age ± SD: 48.1 ± 13.4; 57.7% were males)	Abdominal ultrasound, exclusion of liver disease of other etiology. Additionally: Liver histology in one study. The US-Fatty Liver Index (US-FLI) in one study.	N/A	In the fixed effects model, it was shown that AH was a risk factor for NAFLD (Z = 13.46, *p* < 0.001)

AH = arterial hypertension; BP = blood pressure; CI = confidence interval; DBP = diastolic blood pressure; FIB-4 = fibrosis-4 index; FLI = fatty liver index; N/A = not applicable; NAFLD = non-alcoholic fatty liver disease; OR = odds ratio; SBP = systolic blood pressure; SD = standard deviation; US = ultrasound.

## Data Availability

Not applicable.

## Abbreviations

ACE	angiotensin-converting enzyme
ADIPOQ	adiponectin
AGTR1	angiotensin II receptor type 1
AH	arterial hypertension
AHR	aryl hydrocarbon receptor
Akt	protein kinase B
BMI	body mass index
BP	blood pressure
CI	confidence interval
CVD	cardiovascular disease
DBP	diastolic blood pressure
ET-1	endothelin-1
FFAs	free fatty acids
FGF-21	fibroblast growth factor 21
FIB-4	fibrosis-4 index
FLI	fatty liver index
GPR	G protein-coupled receptor
HDL	high-density lipoproteins
HFF	hepatic fat fraction
HOMA-IR	homeostasis model assessment of insulin resistance
HR	hazard ratio
hsCRP	high-sensitivity C-reactive protein
IL	interleukin
IR	insulin resistance
LDL	low-density lipoproteins
LEP	leptin
MAPK	mitogen-activated protein kinase
MRI	magnetic resonance imagining
N/A	not applicable
NAFLD	non-alcoholic fatty liver disease
NFS	NAFLD fibrosis score
NO	nitric oxide
Olfr	olfactory receptor
OR	odds ratio
PCOS	polycystic ovary syndrome
PERK	protein kinase RNA- like endoplasmic reticulum kinase
PI3K	phosphatidylinositol 3-kinase
RAAS	renin-angiotensin-aldosterone system
RR	risk ratio
SBP	systolic blood pressure
SCFAs	short-chain fatty acids
SD	standard deviation
SUA	serum uric acid
T2DM	type 2 diabetes mellites
TGFB1	transforming growth factor beta-1
TMA	trimethylamine
TMAO	trimethylamine N-oxide
TNF-α	tumor necrosis factor-α
US	ultrasound
VLDL	very-low-density lipoproteins
